# Sequencing and analysis of the complete mitochondrial genome of *Metrioptera bonneti* (Orthoptera: Tettigoniidae)

**DOI:** 10.1080/23802359.2018.1524276

**Published:** 2018-10-27

**Authors:** Fei Liu

**Affiliations:** College of Life Sciences and Food Engineering, Shaanxi Xueqian Normal University, Xi’an, R.P. China

**Keywords:** *Metrioptera bonneti*, mitogenome, phylogeny

## Abstract

Here, we report the complete mitochondrial genome sequence of *Metrioptera bonneti* and its phylogenetic status. The complete mitogenome of *M. bonneti* is 16,273 bp in size and consisted of 13 protein-coding genes, 22 tRNA genes, two rRNA genes, and one D-loop. The overall nucleotide composition of the mitogenome was 35% A, 20.4% C, 11.7% G, and 32.9% T, with an A + T bias of 67.9%. Using the 13 PCGs and two rRNA of *M. bonneti*, together with 39 Tettigoniidae and two outgroup species, we constructed a phylogenetic tree based on the Bayesian inference method. The tree showed that *M. bonneti* forms a sister clade with *Metrioptera ussuriana* and supported the monophyly of Tettigoniinae.

*Metrioptera bonneti* belongs to Tettigoniinae, Tettigoniidae, Orthoptera. The study's specimen of *M. bonneti* was collected from Zhuolu, Hebei, China (N 40°38′, E 115°20′), on 21 August 2005, and was kept in the Molecular and Evolutionary Lab at Shaanxi Normal University. Total DNA was extracted from the leg muscle of the specimen (Zhou et al. 2010), after which the genome was sequenced using next-generation sequencing technology. The HiSeq (Illumina, San Diego, CA) run generated 20,984,772 raw reads containing a total of 3.16 Gb, and the length per read is approximately 150 bp. The low-quality reads were removed using the CLC Genomics Workbench 11.0 (CLC Bio, Aarhus, Denmark), and the mitochondrial genome of *M. bonneti* was assembled using Mira 4.0.2 and MITObim 1.7 (Hahn et al. 2013). The annotation of protein-coding genes (PCGs), rRNA genes and D-loop was conducted using Geneious 10.1.2 (Biomatters Ltd., Auckland, New Zealand). tRNA genes were predicted using the online software, MITOS (Bernt et al. 2013).

The complete mitogenome of *M. bonneti* was found to be 16,273 bp in size and has been deposited in GenBank under the accession number MH685924. The organism’s mitogenome contained 35% A, 20.4% C, 11.7% G, and 32.9% T bases, with an AT bias of 67.9%. Mitogenome analysis also revealed a total of 13 PCGs, 22 tRNA genes, two rRNA genes, and one D-loop. Among the 37 genes, four PCGs (*ND5*, *ND4*, *ND4L* and *ND1*), two rRNA genes, and eight tRNA genes (*tRNA^Gln^*, *tRNA^Cys^*, *tRNA^Tyr^*, *tRNA^Phe^*, *tRNA^His^*, *tRNA^Pro^*, *tRNA^Leu^* and *tRNA^Val^*) are encoded on the N-strand, and the other 23 genes are encoded on the J-strand. Of the 13 identified PCGs, the starting codon for *COX1* was CCG, whereas that for *ND2* was ATC. *ND6*, *ATP8*, *ND3* and *ND5* had ATT as the starting codon, whereas the other seven genes had ATG. Regarding stop sequences, each of *COX1*, *COX2*, *ATP6*, *ND5,* and *ND4* had a single stop nucleotide, T. *ND3* on the other hand ended with TAG, while the other six genes had TAA as the stop codon. The length of the tRNA genes ranged from 62 to 70 bp. The *16S rRNA* and *12S rRNA* genes were 1,310 bp and 782 bp in length respectively, whereas the D-loop was as long as 1,489 bp.

We performed phylogenetic analysis using 42 concatenated mitogenome datasets (13 PCGs and two rRNAs) including the *M. bonneti* data from this study, 39 species from Tettigoniidae and two Caeliferan outgroup species (Figure 1). Using the Bayesian inference method, we found that *M. bonneti* and *Metrioptera ussuriana* (both of which belong to the same genus), were grouped together with a 100% bootstrap support and were sister groups. This finding indirectly verifies the accuracy of the mitochondrial genome sequencing of *M. bonneti*, while the phylogenetic tree also supports the monophyly of Tettigoniinae (Figure 1).

**Figure 1.**
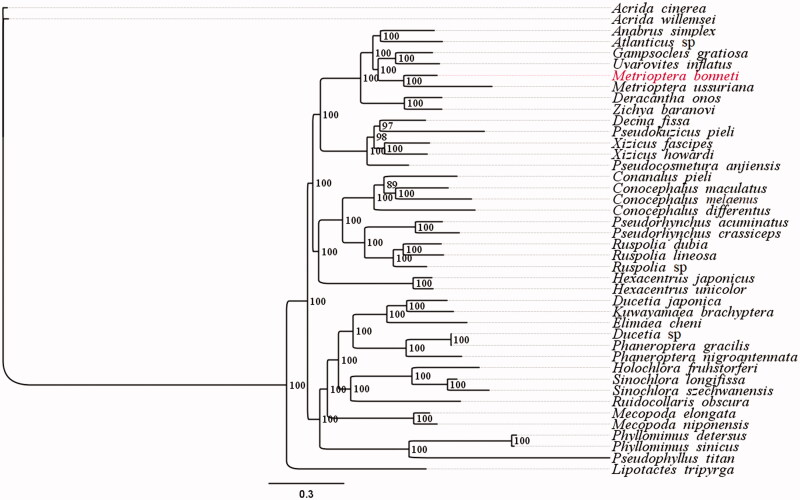
Phylogenetic tree generated based on PCGs and rRNAs using mitochondrial genomes from species in Tettigoniidae. The GenBank accession numbers for tree construction is listed as follows: *Acrida cinerea* (GU344100), *Acrida willemsei* (EU938372), *altbrus simplex* (NC_009967), *Atlanticus* sp. (KX057730), *Gampsocleis gratiosa* (NC_011200), *Uvarovites inflatus* (NC_026231), *Metrioptera ussuriana* (NC_034796), *Deracantha onos* (NC_011813), *Zichya baranovi* (NC_033984), *Decma fissa* (NC_033981), *Pseudokuzicus pieli* (NC_033982), *Xizicus fascipes* (NC_018765), *Xizicus howardi* (KY458226), *Pseudocosmetura anjiensis* (NC_033853), *Conanalus pieli* (NC_033987), *Conocephalus maculatus* (HQ711931), *Conocephalus melaenus* (KY407794), *Conocephalus differentus* (MF347703), *Pseudorhynchus acuminatus* (NC_033992), *Pseudorhynchus crassiceps* (NC_033990), *Ruspolia dubia* (EF583824), *Ruspolia lineosa* (NC_033991), *Ruspolia* sp. (KX057717), *Hexacentrus japonicus* (NC_033983), *Hexacentrus unicolor* (NC_033999), *Ducetia japonica* (KY612457), *Kuwayamaea brachyptera* (NC_028159), *Elimaea cheni* (NC_014289), *Ducetia* sp. (KX673198), *Phaneroptera gracilis* (NC_034756), *Phaneroptera nigroantennata* (NC_034757), *Holochlora fruhstorferi* (NC_033993), *Sinochlora longifissa* (NC_021424), *Sinochlora szechwanensis* (KX354724), *Ruidocollaris obscura* (NC_028160), *Mecopoda elongata* (NC_021380), *Mecopoda niponensis* (NC_021379), *Phyllomimus detersus* (NC_028158), *Phyllomimus sinicus* (NC_033997), *Pseudophyllus titan* (NC_034773) and *Lipotactes tripyrga* (NC_033996).
